# The effect of primary tumor volume on the prognosis of nasopharyngeal carcinoma in era of volumetric modulated arc therapy: a propensity score matched cohort study

**DOI:** 10.1016/j.bjorl.2023.02.002

**Published:** 2023-02-16

**Authors:** Xiang Lin, Bingyi Wang, Fei Zheng, Zhaodong Fei, Chuanben Chen

**Affiliations:** Clinical Oncology School of Fujian Medical University, Fujian Cancer Hospital, Department of Radiation Oncology, Fujian, China

**Keywords:** Primary tumor volume, Prognosis, Nasopharyngeal carcinoma, Propensity score matching, Volumetric modulated arc therapy

## Abstract

**Objective:**

The role of Primary Tumor Volume (PTV) in Nasopharyngeal Carcinoma (NPC) treated with Volumetric Modulated Arc Therapy (VMAT) is still unclear. The aim of this study was to access the effect of PTV in prognosis prediction of nasopharyngeal carcinoma in era of VMAT.

**Methods:**

Between January 20 and November 2011, 498 consecutive NPC patients with stage I–IVA disease who received VMAT at a single center were retrospectively analyzed. Receiver Operating Characteristic (ROC) was performed to access the cut-off point of PTV. Univariate Kaplan-Meier and multivariate Cox regression analyses were used to evaluate prognostic value for PTV. The Propensity Score Matching (PSM) was used to adjust baseline potential confounders.

**Results:**

The 5-year Locol-Regional Failure-Free (L-FFR), Distant Failure-Free Survival (D-FFR), Disease-Free Survival (DFS) and Overall Survival (OS) were 90.6%, 83.7%, 71.5% and 79.3%, respectively. Before PSM, the 5-year L-FFR, D-FFR, DFS, OS rates for NPC patients with PTV ≤ 38 mL vs. PTV > 38 mL were 94.1% vs. 90.4% (*p* = 0.063), 87.9% vs. 76.3% (*p* < 0.001), 78.5% vs. 58.5% (*p* < 0.001) and 86.3% vs. 66.7% (*p* < 0.001) respectively. Multivariate analysis showed PTV was an independent prognostic factor for D-FFS (*p* = 0.034), DFS (*p* = 0.002) and OS (*p* = 0.001). PTV classified was still an independent prognostic factor for OS after PSM (HR = 2.034, *p* = 0.025.

**Conclusions:**

PTV had a substantial impact on the prognosis of NPC patients treated with VMAT before and after PSM simultaneously. PTV > 38 mL may be considered as an indicator of the clinical stage of nasopharyngeal carcinoma.

**Level of evidence:**

III.

## Highlights

• Propensity score matching was used to reduce bias in the study.
• PTV was an important independent predictor on survival for NPC patients receiving VMAT.
• PTV > 38 mL may be considered as an indicator of the clinical stage of nasopharyngeal carcinoma.

## Introduction

Nasopharyngeal Carcinoma (NPC) is a kind of malignant Head and Neck Cancer (HNC) arising from the nasopharynx and has a specific geographic distribution, primarily in Southern China and Southeast Asia.^1^, ^2^ The major treatment modality for NPC is radiotherapy. Due to its quicker treatment time and dosimetric advantages, Volumetric Modulated Arc Therapy (VMAT), a novel type of Intensity-Modulated Radiation Therapy (IMRT), has gained widespread acceptance in place of IMRT for NPC.^3^, ^4^, ^5^, ^6^

The effect of Primary Tumor Volume (PTV) on outcome for NPC has drawn considerable attention in the pass decades. Numerous studies confirmed that PTV was an outstanding prognostic factor for NPC in era of IMRT.^7^, ^8^, ^9^, ^10^ Additionally, some studies suggested that PTV may be included into the TNM staging system and guided therapy selection.^11^, ^12^ However, most of the previous were based on IMRT. Very few studies have evaluated the prognostic value of PTV for NPC patients undergoing VMAT. Nowadays VMAT was widely used in the treatment of NPC, therefore further discussion should be proposed on whether PTV could affect treatment outcome under the condition of VMAT. Hence, the aim of the study was to investigate the effect of primary tumor volume on long-term survival in the NPC patients treated with VMAT.

## Methods

### Patients

Between January 2010 and November 2011, 508 consecutive newly diagnosed NPC patients were treated primarily with VMAT at our center. The inclusion criteria for this retrospective study were: 1) Stage I‒IVA disease in accordance to the 8th edition of the AJCC/UICC TNM staging system; 2) Biopsy-proven; 3) Treated with VMAT. The exclusion criteria were as follows: 1) Diagnosed with a previous malignancy or other concurrent malignancy; 2) Pregnancy or lactation; 3) Metastatic disease at the time of diagnosis. Based on the criteria, 498 patients were included. The characteristics of patients are listed in Table 1. This study was approved by the ethics committee of our center. The participants were contacted by phone to acquire verbal informed consent for retrospective analysis of the relevant clinical data. We also de-identified all patient information.

**Table 1:** Patients’ characteristics.

Characteristic	Number of patient (%)
Age (years)	
≤50	322 (64.7%)
>50	176 (35.3%)
Gender	
Male	383 (76.9)
Female	115 (23.1%)
Kps score	
≤80	19 (3.8%)
90	479 (96.2%)
Radiotherapy Interruption (days)	
<5	454 (91.2%)
≥5	44 (8.8%)
Boost	
Yes	151 (30.3%)
No	347 (69.7%)
T-stage	
T1	39 (7.8%)
T2	149 (29.9%)
T3	214 (43.0%)
T4	96 (19.3%)
N-stage	
N0	127 (25.5%)
N1	242 (48.6%)
N2	106(21.3%)
N3	23 (4.6%)
TNM stage	
I	11 (2.2%)
II	128 (25.7%)
III	248 (49.8%)
IV	111 (22.3%)
Con-CT	
Yes	122 (24.5%)
No	376 (75.5%)
PTV	
≤38 mL	321 (64.5%)
* >*38 mL	177 (35.5%)

Con-CT, Cncurrent Chemotherapy; PTV, Primary Tumor Volume.

### PTV measurement

Contrast enhanced computed tomography scans were performed before treatment of 3 mm slices from the top of the head to 2 cm below the sternoclavicular joint. Image data were imported into a Three-Dimensional (3D) treatment planning system. The PTV was manually outlined slice-by-slice based on fused images of the pretreatment MR images and CT images to access more accurate delineation. The PTV, including the primary tumor and retropharyngeal node, was automatically calculated by reconstruction a 3D image.

### Radiotherapy

All the patients included in this study were treated with VMAT. The target volumes were outlined manually according to our treatment protocol defined in previous studies.^13^, ^14^ The primary Gross Tumor Volume (GTV-P) and the involved lymph Nodes (GTV-N) included all gross disease as determined by imaging, clinical and endoscopic findings. The Clinical Target Volumes (CTVs) were designed to encompass microscopic disease including the high-risk regions (CTV-1) and the low-risk regions (CTV-2). The CTV-1 was defined as the high-risk region that included GTV plus 5–10 mm margin, including the nasopharyngeal mucosa (5-mm submucosal volume). The CTV-2 was designed for subclinical prophylactic low-risk region. Levels II, III, IV, and V can be incorporated into Clinical Target Volume of the Neck nodal regions (CTV-N), as recommended by the RTOG delineation consensus for head and neck malignancies. The planning target volume was created based on each volume with an additional 3-mm margin, allowing for setup variability. OARs include the brain stem, spinal cord, optic nerve, optic chiasm, temporal lobe, crystal, parotid, pituitary, mandibular and so on.

A total dose of 69.7‒70 Gy/31‒35 fractions at 2‒2.25 Gy/fraction was set to the planning target volume of GTV-P and GTV-N. While 59.5‒62 Gy at 1.7‒2 Gy/fraction was prescribed to the PTV of CTV-1 and 52.7‒56 Gy at 1.6‒1.8 Gy/fraction was prescribed to planning target volume of CTV-2 and CTV-N. 151 patients with residual disease including primary tumor and metastatic regional lymph nodes observed by imaging or nasopharyngoscope after radiotherapy received a boost of 4‒12 Gy by VMAT.

### Chemotherapy

According to NCCN guidelines, 2–4 cycles of cisplatin-based neoadjuvant chemotherapy, along with 1–2 cycles of concurrent chemotherapy, were used to patients with stage III‒IV disease. Patients suffered from stage T1-2N1 disease received 1–2 cycles of concurrent chemotherapy. Patients at stage T1-2N0 only subjected to radiation. Neoadjuvant chemotherapy included gemcitabine (1000 mg/m^2^ on days 1 and 8) plus cisplatin (80 mg/m^2^ on days 1‒3) or paclitaxel (135 mg/m^2^ on day 1) plus cisplatin (80 mg/m^2^ on days 1‒3). Concurrent chemotherapy based on cisplatin (80 mg/m^2^ on days 1‒3) was administered during radiotherapy. Concurrent and adjuvant chemotherapy were repeated every 21 days.

### Follow-up

All the patients were followed up trimonthly after radiotherapy completion for the first 2-years, every 6-months for the next 3-years, and annually thereafter. During follow-up, complete physical, hematologic, and biochemical examinations, chest radiograph, abdominal ultrasonography, and nasopharyngoscope examination were included. MRI/CT of the nasopharynx was performed every 6‒12 months. All local or regional relapse was confirmed by pathology.

### Statistical methods

Statistical analysis was performed using SPSS statistical software version 22.0 (SPSS Inc.Chicago, IL). Receiver Operating Characteristic (ROC) curve analysis was performed to explore the optimal cut-off point of PTV to predict mortality. The Area Under the ROC Curve (AUC) was used to identify the prognostic value of PTV. After then, the population of the study was divided into two groups based on the ideal cut-off point. The Propensity Score Matching (PSM) was performed for 1:1 matching to reduce possible selection bias between the two groups. A caliper of 0.05 was set to enhance the matching quality.

Survival outcomes, including 5-year Local Failure-Free Rate (L-FFR), 5-year Distant Failure-Free Rate (D-FFR), 5-year Disease-Free Survival (DFS) and 5-year Overall Survival (OS), were calculated from the time of diagnosis to the most recent follow-up or to the date of recurrence, metastasis, or death. The Kaplan-Meier method was used for survival analysis. The log-rank test was used to calculate the significance of differences between multiple survival curves. Cox proportional hazards regression analysis was used to assess the independent significance of different prognostic factors. Two-sided p-values < 0.05 were accepted as statistical significance.

## Results

### Treatment outcomes

With a median follow-up of 68 months (range, 4‒110 months), 47 patients had local or regional failures, 81 patients had distant metastasis, and 10 patients had distant metastasis with local or regional failures. The 5-year locol-regional failure-free, distant failure-free survival, disease-free survival and overall survival rates were 90.6%, 83.7%, 71.5% and 79.3% respectively.

### The optimal cut-off points of PTV

ROC curve analysis was conducted to confirm the best cut-off point of PTV. The optimal cut-off point of PTV was 38.15 mL (sensitivity: 57.7%; specificity: 70.2%). The area under the ROC curve was 0.636 (95% CI 0.573–0.698; *p* < 0.001). Hence, we selected the cut-off point as 38 mL to divide patients into PTV ≤ 38 mL group and PTV > 38 mL group for survival analysis.

### Baseline characteristics before and after PSM

The baseline characteristics of the 498 patients are shown in Table 1. According to the cut-off point, 321 patients were categorized as PTV ≤ 38 mL group and 177 patients were categorized as PTV > 38 mL group. Significant differences were observed between the two groups before PSM with respect to gender (*p* < 0.001), age (*p* < 0.001), N-stage (*p* = 0.046), T-stage (*p* < 0.001), KPS score (*p* < 0.001), boost (*p* = 0.035). Therefore, PSM in a ratio of 1:1 with a caliper of 0.05 without replacement was carried out to reduce the confounding bias between the two groups using the following baseline characteristics: age, gender, KPS score, radiotherapy interruption, boost, T-stage, N-stage, TNM-stage, concurrent chemotherapy. Ultimately, 103 pairs of patients were generated. Baseline characteristics were well balanced between the two matched groups (Table 2).

**Table 2:** Baseline characteristics of PTV ≤ 38 mL group and PTV > 38 mL group before and after PSM.

Variable		Before PSM			After PSM		
		PTV ≤ 38 mL	PTV > 38 mL	*p*-value	PTV ≤ 38 mL	PTV > 38 mL	*p-*value
Gender	Male	232 (72.2)	151 (85.3)	0.001	79 (76.7)	83 (80.6)	*0.497*
	Female	89 (27.8)	26 (14.7)		24 (23.3)	20 (19.4)	
Age	≤50 y	116 (36.1)	118 (66.7)	<0.001	68 (66.0)	74 (71.8)	0.366
	>50 y	205 (63.9)	59 (33.3)		35 (34.0)	29 (28.2)	
N-stage	N0	82 (25.5)	45 (25.4)	0.046	28 (27.2)	24 (23.3)	0.830
	N1	168 (52.3)	74(41.8)		43 (41.7)	41 (39.8)	
	N2	57 (17.8)	49 (27.7)		31 (30.1)	37 (35.9)	
	N3	14 (4.4)	9 (5.1)		1 (1.0)	1 (1.0)	
T-stage	T1	35 (10.9)	4 (2.3)	<0.001	4 (3.9)	4 (3.9)	0.571
	T2	131 (40.8)	18 (10.2)		14 (13.6)	18 (17.5)	
	T3	132 (41.1)	82 (46.3)		65 (63.1)	68 (66.0)	
	T4	23 (7.2)	73(41.2)		20 (19.4)	13 (12.6)	
KPS score	≤80	4 (0.9)	15 (8.4)	<0.001	2 (2.0)	3 (3.0)	1.000
	90	318 (99.1)	162 (91.6)		101 (98.0)	100 (97.0)	
Con-CT	Yes	70 (21.8)	52 (29.4)	0.06	29 (28.2)	35 (34.0)	0.366
	No	251 (78.2)	125 (70.6)		74 (71.8)	68 (66.0)	
Int	Yes	19 (5.9)	15 (8.5)	0.279	8 (7.8)	9 (8.7)	1.000
	No	302 (94.1)	162 (91.5)		95 (92.2)	94 (91.3)	
Boost	Yes	87 (27.1)	64 (36.2)	0.035	35 (34.0)	37(	0.770
	No	234 (72.9)	113 (63.8)		68 (66.0)	66 (64.0)	

PSM, Propensity Score Matching; Con-CT, Concurrent Chemotherapy; PTV, Primary Tumor Volume.

### Survival analysis

Age, gender, N-stage, T-stage, Primary Tumor Volume (PTV), KPS score, boost, interruptions during radiotherapy, and concurrent chemotherapy were included in the survival analysis. The results of univariate analysis were shown in Table 3. Multivariate analysis in the original unmatched cohort showed age (Hazard Ratio [HR = 2.318, *p* < 0.001], N-stage [HR =  1.941, *p* = 0.001], PTV [HR = 2.298, *p* < 0.001] were adverse prognostic factors for OS (Table 4). All the factors were not independently prognostic for L-FFR. N-stage (HR = 2.814, *p* < 0.001) and PTV (HR = 1.671, *p* = 0.034) were adverse prognostic factors for D-FFS. Age (HR = 1.853, *p* < 0.001), N-stage (HR = 1.954, *p* = 0.001) and PTV (HR = 1.772, *p* = 0.002) were adverse prognostic factors for DFS. A total of 206 matched patients were eligible for further analysis after PSM. Multivariate analysis revealed that age (HR = 2.375, *p* = 0.004) and PTV (HR = 2.034, *p* = 0.025) were still independent factors affecting OS (Table 5).

**Table 3:** Univariate analysis of predictive factors for the patients with NPC.

Variable		5-year L-FFR (%)	*p*-value	5-year D-FFS (%)	*p*-value	5-year DFS (%)	*p*-value	5-year OS (%)	*p*-value
Gender	Male	90.4	0.671	83.1	0.186	70.4	0.063	78.7	0.128
	Female	91.3		87.8		78.3		82.6	
Age	≤50 y	92.3	0.037	84.3	0.735	76.9	0.001	84.6	<0.001
	>50 y	87.5		84.1		63.6		70.5	
N-stage	N0	94.5	0.257	91.3	<0.001	81.9	<0.001	86.6	0.004
	N1	89.3		88.5		74.5		81.1	
	N2	89.6		68.9		57.5		70.8	
	N3	87.5		70.8		62.5		66.7	
T-stage	T1	92.3	0.336	92.3	0.031	84.6	<0.001	94.9	<0.001
	T2	91.9		87.2		79.2		85.2	
	T3	91.1		84.1		71.5		79.4	
	T4	86.7		76.5		58.2		65.3	
KPS score	≤80	94.7	0.761	73.7	0.189	52.6	0.054	63.2	0.105
	90	92.7		84.1		72.2		80.0	
Con-CT	Yes	90.2	0.735	85.7	0.106	73.3	0.218	79.5	0.895
	No	90.7		79.5		68.9		79.3	
Int	Yes	90.1	0.193	84.1	0.860	79.4	0.397	79.4	0.697
	No	97.1		85.3		71.7		82.4	
Boost	Yes	91.7	0.218	85.6	0.210	64.5	0.025	73.0	0.030
	No	88.2		80.9		75.6		82.5	
PTV	≤38 mL	94.1	0.063	87.9	<0.001	78.5	<0.001	86.3	<0.001
	>38 mL	90.4		76.3		58.5		66.7	

Neo-CT, Neoadjuvant Chemotherapy; Con-CT, Concurrent Chemotherapy; Int, Interruption during radiotherapy; 5-y L-FFR, 5-year Locol-regional Failure-Free Rate; 5-y D-FFS, 5-year Distant Failure-Free Survival; 5-y DFS, 5-year Disease Free Survival; 5-y OS, 5-year Overall Survival; PTV, Primary Tumor Volume.

**Table 4:** Multivariate analysis of predictive factors for the patients with NPC before PSM.

Variable	5-year L-FFR		5-year D-FFS		5-year DFS		*5-year OS*	
	HR (95% CI)	*p*	HR (95% CI)	*p*	HR (95% CI)	*p*	HR (95% CI)	*p*
Age	1.681 (0.863‒3.273)	0.126	1.155 (0.727‒1.835)	0.542	1.853 (1.326‒2.590)	<0.001	2.318 (1.568‒3.425)	<0.001
N-stage								
N0‒1 vs. N2‒3	1.393 (0.678‒2.862	0.367	2.814 (1.803‒4.392)	<0.001	1.954 (1.381‒2.767)	0.001	1.941 (1.296‒2.908)	0.001
T-stage								
T1‒2 vs. T3‒4	1.977 (0.859‒4.547)	0.109	1.413 (0.834‒2.394)	0.199	1.414 (0.948‒2.111)	0.090	1.530 (0.938‒2.498	0.089
PTV								
≤38 mL vs. >38 mL	1.452 (0.720‒2.928)	0.297	1.671 (1.040‒2.686	0.034	1.772 (1.237‒2.539	0.002	2.298 (1.504‒3.512)	<0.001
* Con-CT*	0.985 *(*0.456*‒*2.130*)*	0.969	0.818 *(*0.508*‒*1.318*)*	0.410	0.895 *(*0.614*‒*1.305*)*	0.583	1.081 *(*0.683*‒*1.712*)*	0.739

PSM, Propensity Score Matching; HR, Hazard Ratio; CI, Confidence Interval; Con-CT, Concurrent Chemotherapy;5-y L-FFR, 5-year Locol-regional Failure-Free Rate; 5-y D-FFS, 5-year Distant Failure-Free Survival; 5-y DFS, 5-year Disease Free Survival; 5-y OS, 5-year Overall Survival; PTV, Primary Tumor Volume.

**Table 5:** Multivariate analysis of predictive factors for the patients with NPC after PSM.

Variable	5-year L-FFR		5-year D-FFS		5-year DFS		*5-year OS*	
	HR (95% CI)	*p*	HR (95% CI)	*p*	HR (95% CI)	*p*	HR (95% CI)	*p*
Age	0.874 (0.235‒3.249)	0.841	1.315 (0.686‒2.520)	0.409	1.832 (1.106‒3.036)	0.019	2.375 (1.309‒4.309)	0.004
N-stage								
N0‒1 vs. N2‒3	1.092 (0.328‒3.639)	0.886	2.275 (1.226‒4.223)	0.009	1.552 (0.935‒2.576)	0.089	1.708 (0.933‒3.124)	0.083
T-stage								
T1‒2 vs. T3‒4	2.750 (0.350‒6.601	0.336	1.326 (0.582‒3.021)	0.501	1.433 (0.723‒2.837)	0.302	1.517 (0.670‒3.432)	0.317
PTV								
≤38 mL vs. >38 mL	1.110 (0.355‒3.468)	0.858	1.293 (0.693‒2.411)	0.419	1.408 (0.852‒2.329)	0.182	2.034 (1.092‒3.786)	0.025
Con-CT	0.938 *(*0.278*‒*3.157*)*	0.917	1.192 *(*0.602*‒*2.359*)*	0.614	0.987 *(*0.577*‒*1.691*)*	0.963	1.354 *(*0.678*‒*2.704*)*	0.390

PSM, Propensity Score Matching; HR, Hazard Ratio; CI, Confidence Interval; Con-CT, Concurrent Chemotherapy;5-y L-FFR, 5-year Locol-regional Failure-Free Rate; 5-y D-FFS, 5-year Distant Failure-Free Survival; 5-y DFS, 5-year Disease Free Survival; 5-y OS, 5-year Overall Survival; PTV, Primary Tumor Volume.

### Prognostic value of the PTV cut-off point before and after PSM

**Figure 1:**
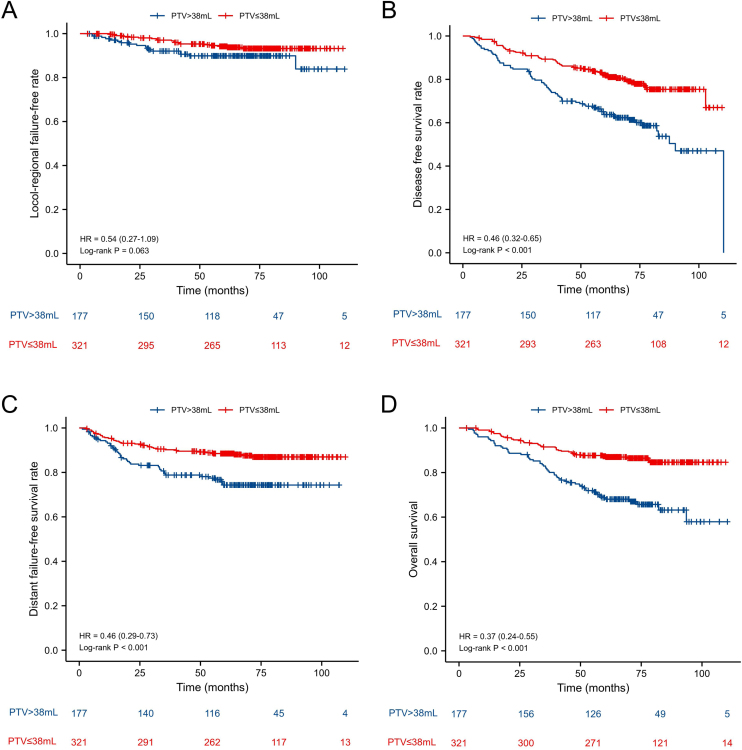
Kaplan-Meier curves for locol-regional failure-free (A), disease free survival (B), distant failure-free survival (C) and overall survival (D) between PTV > 38 mL group and PTV ≤ 38 mL group before propensity score matching.

**Figure 2:**
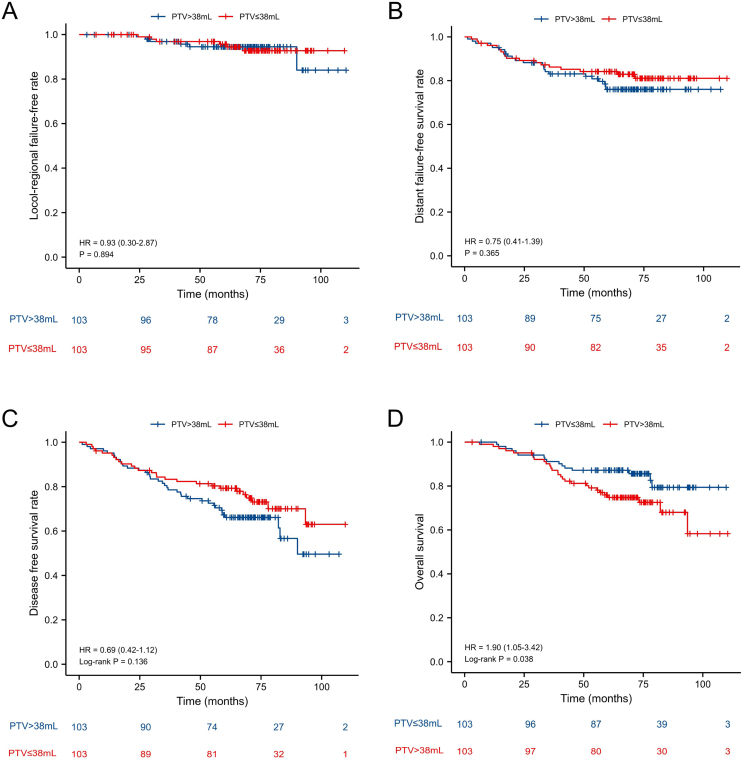
Kaplan-Meier curves for locol-regional failure-free (A), distant failure-free survival (B), disease free survival (C) and overall survival (D) between PTV > 38 mL group and PTV ≤ 38 mL group after propensity score matching.

In all patients before PSM, the 5-year L-FFR, D-FFR, DFS, OS rates for NPC patients with PTV ≤ 38 mL vs. PTV > 38 mL were 94.1% vs. 90.4% (*p* = 0.063), 87.9% vs. 76.3% (*p* < 0.001), 78.5% vs. 58.5% (*p* < 0.001) and 86.3% vs. 66.7% (*p* < 0.001) respectively (Fig. 1). In multivariate analysis, PTV using the cut-off point was an independent prognostic factor for D-FFS (*p* = 0.034), DFS (*p* = 0.002) and OS (*p* = 0.001). After PSM, the OS of PTV ≤ 38 mL group was better than that of the other group (HR = 1.90, *p* = 0.038). While the L-FFR, D-FFR and DFS were comparable between the two groups (Fig. 2). On multivariable analysis, PTV classified by the cut-off point was still an independent prognostic factor for OS (HR = 2.034, *p* = 0.025, Table 4).

## Discussion

With the emergence of VMAT, the treatment strategy for NPC has greatly improved. VMAT has been more widely utilized in the treatment of NPC. Most previous studies are merely limited to conventional radiotherapy and IMRT to investigate the prognostic relevance of PTV in NPC patients. It is not sufficient apparently. Thus, we conducted the study to explored the prognostic value of PTV based on VMAT. Furthermore, PSM was applicated to reduce possible selection bias. To the best of our knowledge, this is the first study to address the prognostic value of PTV in NPC patients undergoing VMAT.

Many studies have revealed beneficial results of IMRT for the NPC treatment. Nonetheless, there are some limitations of IMRT without a doubt. Longer treatment duration of IMRT may limit radiotherapy precision due to increased intra-fractional patient movements.^15^ In addition, the incidence of radiation-induced secondary cancers may be increased because of the higher dose to OARs of IMRT compared with VMAT.^16^, ^17^, ^18^ A number of recent studies have shown that VMAT could provide dosimetric advantages over IMRT in treatment plan to some extent.^3^, ^4^, ^5^, ^19^ Therefore, VMAT has been widely adopted to replace IMRT gradually because of quicker treatment duration and dosimetric advantages. Thus, it is reasonable to reevaluate the existing prognostic value of PTV in the era of VMAT.

PTV has attracted extensive attention over recent years. The impact of PTV on the treatment outcome of NPC patients has been previously studied. Ni et al.^10^ reported that the primary gross tumor volume was an independent prognostic factor for local control of NPC patients. A retrospective study conducted by Qin et al.^20^ indicated that advanced NPC patients with PTV ≥ 33 mL had poorer OS, locoregional control and more distant metastasis than patients with PTV < 33 mL. Similar results were shown in our previous study.^11^ However, the radiotherapy techniques of these previous trails were IMRT. In the present study we just focused on the NPC patients undergoing VMAT. In consistent with previous results, univariate analysis of our current study indicated that PTV was significantly associated with D-FFR (*p* < 0.001), DFS (*p* < 0.001) and OS (*p* < 0.001). Multivariate analysis results showed that PTV had significant influences on D-FFS (*p* = 0.034), DFS (*p* = 0.002) and OS (*p* < 0.001) respectively. Furthermore, PSM was performed to reduce the confounding bias in the cohort. After PSM, PTV still was a crucial prognostic factor for OS on multivariate analysis.

The possible reasons are as followed. Some adverse biological factors, including hypoxia, radio resistance and the number of tumors clonogen, may be related to poor overall survival rate of larger tumor volume. It is usually accepted that oxygenation is of paramount importance for the efficacy of radiation therapy. More clonogen cells will exist with tumor volume increasing and the sensitivity of the cloned cells responding to radiotherapy is low in large tumor, which will lead to more difficulties in the treatment.^21^ In addition, it is considerably possible to find tumor with larger volume close to critical organs such as brainstem which may significantly influence dose coverage of target volume and curative effect ultimately.

Unfortunately, the cut-off points of PTV to categorize patients into good and poor prognostic groups is still controversial. Chen et al.^11^ used four categories of PTV (≤15, 15–25, 25–50 and >50 mL) and demonstrated a significant difference in overall survival with a PTV > 50 mL. Qin et al.^20^ demonstrated that 33 mL was determined as the cut-off point of PTV for advanced NPC patients. Wu et al.^22^ proposed that 25 mL of PTV was the cut-off point for the probability of distant metastasis. In the current investigation, ROC analysis was performed in an effort to access the critical PTV. We established the essential cut-off point based on the maximum Youden score and clinical applicability. Thus, a PTV cut-off value of 38 mL was selected for predicting the treatment outcomes of NPC undergoing VMAT. Our data suggested patients with a PTV > 38 mL had worse D-FFS, DFS and OS than patients with a PTV ≤ 38 mL. Even after PSM, PTV > 38 mL was significantly associated with poorer OS. The variations in PTV cut-off points may be attributed to the differences of the patients and radiotherapy techniques. Patients enrolled from single institution in the most studies and selection bias could not be excluded completely. In the study, patients were well balanced after PSM and the only radiotherapy was VMAT. Hence, the PTV cut-off point of 38 mL may be appropriate in the era of VMAT.

Currently, the TNM staging system developed by UICC is the most widely used to predict the prognosis of patients with NPC. Our study showed that N stage was an independent prognostic factor affecting D-FFS, DFS and OS, while T-stage could not affect treatment outcomes for NPC patients treated with VMAT. The results indicated that the current TNM staging system had limitations in its ability to predict treatment outcomes for NPC patients, which were consistent with previous reports.^11^, ^12^ It has become extremely important to search for effective indicators to evaluate prognosis for NPC patients undergoing VMAT. As for PTV, in comparison to NPC patients with a PTV ≤ 38, those with a PTV > 38 mL had worse OS in the research. The conclusion was also still valid after PSM. We proposed that PTV may be considered as a stage indicator of the clinical stage of NPC so that prognostic assessment could be improved. Current treatment strategy may be intensified for the NPC patients with a large PTV to access better treatment outcomes, even though in the early T stage. Moreover, existing treatment intensity for NPC patients with a small PTV may be reduced to avoid over-treatment, particularly without lymphatic metastasis. Further research are needed to explore the optimal treatment strategy.

Despite our positive findings, there are still some limitations that must be addressed in our research. First, the principal limitation of the study is its retrospective nature. Second, it is a single-center study and due caution should be exercised while interpreting and extrapolating the results to other populations. A prospective multi-centered study is warranted for further investigation to provide more definitive evidence.

## Conclusions

PTV is a potential prognostic predictor in NPC patients receiving VMAT. PTV may be considered as an additional stage indicator in the new revision of the clinical stage of NPC. Efforts should be made to conform our findings and investigate individualized treatment strategies to improve the treatment outcome of NPC patients treated with VMAT in the future.

## Funding

This research was supported by Startup Fund for scientific research, Fujian Medical University (grant nº 2018QH1230), the National Clinical Key Specialty Construction Program, Fujian Provincial Clinical Research Center for Cancer Radiotherapy and Immunotherapy (Grant nº 2020Y2012).

## Conflicts of interest

The authors declare no conflicts of interest.
